# Evaluation of the Bioelectrochemical Approach and Different Electron Donors for Biological Trichloroethylene Reductive Dechlorination

**DOI:** 10.3390/toxics10010037

**Published:** 2022-01-13

**Authors:** Edoardo Dell’Armi, Marta Maria Rossi, Lucia Taverna, Marco Petrangeli Papini, Marco Zeppilli

**Affiliations:** Department of Chemistry, Sapienza University of Rome, Piazzale Aldo Moro 5, 00185 Rome, Italy; martamaria.rossi@uniroma1.it (M.M.R.); taverna.1701028@studenti.uniroma1.it (L.T.); marco.petrangelipapini@uniroma1.it (M.P.P.); marco.zeppilli@uniroma1.it (M.Z.)

**Keywords:** chlorinated aliphatic hydrocarbons, reductive dechlorination, bioelectrochemical systems

## Abstract

Trichloroethylene (TCE) and more in general chlorinated aliphatic hydrocarbons (CAHs) can be removed from a contaminated matrix thanks to microorganisms able to perform the reductive dechlorination reaction (RD). Due to the lack of electron donors in the contaminated matrix, CAHs’ reductive dechlorination can be stimulated by fermentable organic substrates, which slowly release molecular hydrogen through their fermentation. In this paper, three different electron donors constituted by lactate, hydrogen, and a biocathode of a bioelectrochemical cell have been studied in TCE dechlorination batch experiments. The batch reactors evaluated in terms of reductive dechlorination rate and utilization efficiency of the electron donor reported that the bio-electrochemical system (BES) showed a lower RD rate with respect of lactate reactor (51 ± 9 µeq/d compared to 98 ± 4 µeq/d), while the direct utilization of molecular hydrogen gave a significantly lower RD rate (19 ± 8 µeq/d), due to hydrogen low solubility in liquid media. The study also gives a comparative evaluation of the different electron donors showing the capability of the bioelectrochemical system to reach comparable efficiencies with a fermentable substrate without the use of other chemicals, 10.7 ± 3.3% for BES with respect of 3.5 ± 0.2% for the lactate-fed batch reactor. This study shows the BES capability of being an alternative at classic remediation approaches.

## 1. Introduction

Chlorinated aliphatic hydrocarbons (CAHs) are common soil and groundwater contaminants due to their large-scale use and inadequate disposal or storage [1,2,3,4,5]. Over the last 40 years, several technologies have been developed to remove this type of contaminant from the subsoil and groundwater. It is necessary to develop efficient technologies specially to achieve the objectives of concentration imposed by national legislation. For example, in Italy, regarding the CAHs of our interest, the threshold concentration limits of perchloroethylene (PCE), trichloroethylene (TCE), 1,1-dichloroethylene (1,1-DCE), and vinyl chloride (VC) are 1.1, 1.5, 0.05 and 0.5 μg/L respectively. 1,2 DCE is classified as non-cancerogenic; with a limit value of 60 μg/L [6].

As new sustainable and eco-friendly strategies are gaining attention [7], bioremediation technologies have been of increasing interest to the science community [8,9,10]. These technologies are based on the activity of specific microorganisms, whose metabolism can transform the contaminant into less toxic and hazardous compounds [11]. For instance, the most important microorganism involved in the degradation of chlorinated compounds is *Dehalococcoides mccarty* (*Dhc*), which can eliminate chlorine atoms (by the reductive dechlorination (RD) pathway) from the carbon skeleton of perchloroethylene (PCE), trichloroethylene (TCE) up to the formation of ethylene, a completely harmless molecule [12]. In the RD reaction, where the direct electron donor is the molecular hydrogen, one chlorine atom is eliminated by inserting two electrons and one hydrogen atom [13]. Since the direct utilization of molecular hydrogen may not be a feasible strategy under field conditions, hydrogen supply is usually provided by the fermentation of more complex substrates, such as ethanol, lactic, butyric, or other volatile fatty acids in general [14,15]. Today, renewable, and more sustainable fermentable substrates have been developed and evaluated in the field [16,17], such as biological-based polyesters, e.g., polyhydroxyalkanoates (PHA) [18,19,20]. However, electron donor injection presents undesirable side-effects due to a large number of organic substrates that may lead to further reactions in the aquifer, consequently deteriorating the quality of the water [21,22]. In this scenario, bio-electrochemical systems (BES) should be a promising technology, notably microbial electrolysis cells (MEC) [23,24,25,26]. BES is well-known electrochemical device wherein certain types of microorganisms may interact with a solid electrode [27,28,29,30]. In particular, the bio-cathode produces hydrogen in concentrations suitable for the proceeding of the biological RD. Recently, different MEC configurations were developed for bioremediation applications, with results that make them applicable on a full scale [31,32,33,34]. In this work, a *Dhc*-enriched culture was tested by investigating three types of electron donors release, which corresponded to lactate, hydrogen, and a bioelectrochemical configuration. The BES system performance was evaluated according to the RD rate (expressed as equivalents) and in terms of electron donor dechlorinating efficiency. Moreover, possible abiotic pathways for TCE removal were verified by carrying out a parallel electrochemical system with the same configuration, but without dechlorination inoculum in the cathodic chamber. Finally, we conducted a comparison with the case studies in the literature (full-scale application) to highlight the differences with this work’s results.

## 2. Materials and Methods

### 2.1. Dehalococcoides Mccartyi (Dhc)-Enriched Culture as Inoculum

All the experiments were carried out by PCE fed dechlorinating anaerobic culture, which was composed of 75% of *D. mccaryi*. Raw data of the 16S rRNA gene amplicon sequencing of the consortium is available at the DDBJ/ENA/GenBank under the BioProject PRJNA705054 (SRA: SRX10172732). This active dechlorinating biomass was used as the inoculum of each reactor as described in the following paragraphs.

### 2.2. Lactate-Fed and H_2_-Fed Anaerobic Cultures

**Figure 1:**
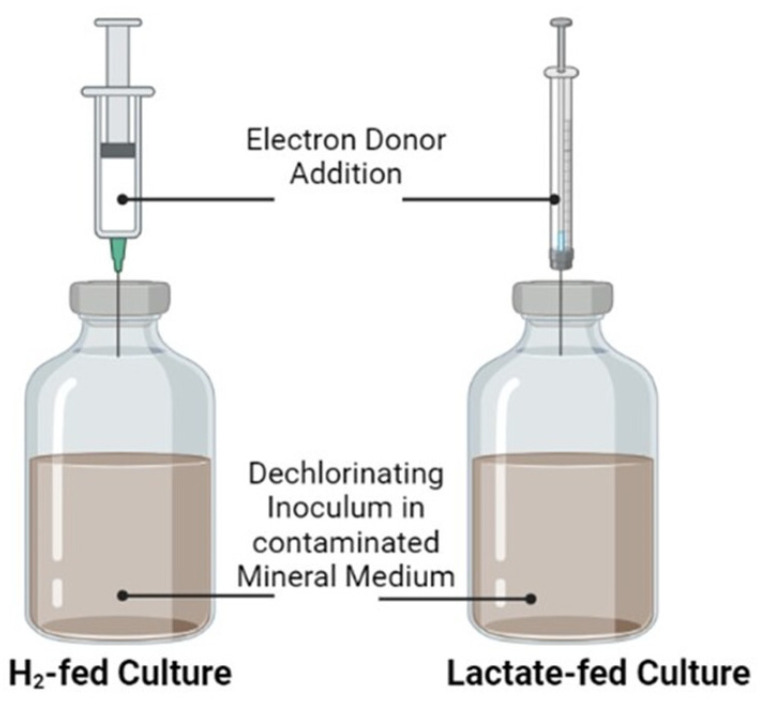
The schematic draw of the batch reactors under different electron donor feeding conditions.

Two batch bioreactors inoculated with the *Dhc*-enriched culture were carried out under “Fill&Draw” conditions using two different electron donors, lactate (named lactated-fed) and H_2_ (indicated with H_2_-fed) (Figure 1). The reactors consisted of a 0.240 L serum bottle where the liquid phase (0.200 L) was composed of inoculum and anaerobic mineral medium solution. The composition of the mineral medium solution was 1 g/L NaCl, 0.048 g/L Na_2_S, 2.52 g/L NaHCO_3_, 0.3 g/L NH_4_Cl, 0.2 g/L KH_2_PO_4_, 0.5 g/L MgCl_2_·6H_2_O, 0.015 g/L CaCl_2_·2 H_2_O, and 1 ml/L of metals solution and 10 mL/L of vitamin solution [24]. For the lactate-fed culture, TCE and 5% *w*/*v* lactate were added as electron acceptor and electron donor, respectively. In parallel, the H_2_-fed anaerobic culture was carried out with the same reactor configuration. In that case, hydrogen gas was injected into the reactor.

The “Fill&Draw” conditions were realized with a hydraulic retention time (HRT) of 56 days. Every 7 days, 25 mL of liquid phase was withdrawn by fresh mineral medium, and the products of the biological RD reaction were removed by flushing the liquid phase with a mixture of N_2_/CO_2_ (70:30) [35]. This procedure has also created an anaerobic environment. The reactor was finally tightly closed with a Teflon butyl cap (Wheaton, Millville, NJ, USA) and an aluminum cap, before the addition of TCE (7 µL) and electron donor.

### 2.3. Electrochemical Systems Setup

**Figure 2:**
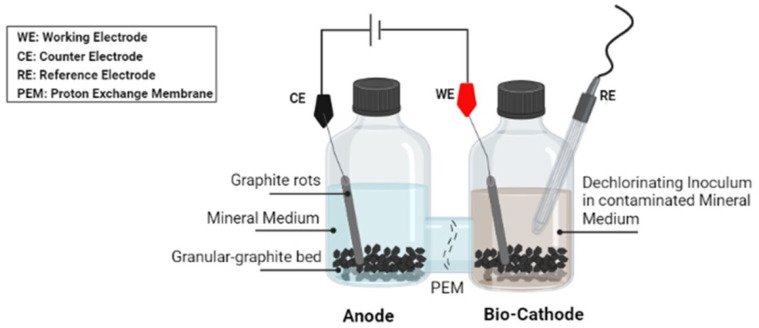
The schematic draw of the “Biotic H-cell” reactor.

Two electrochemical systems were carried out to compare how biotic and abiotic processes behave. The electrochemical systems consisted of a borosilicate H-cell with two 0.240 L compartments, the anode, and the cathode, connected by a lateral flange. A Nafion^®^ (DuPont) proton exchanger membrane (PEM) was used to divide each electrode compartment. A three-electrode set-up was realized [36] and a graphite granular bed was created within the two compartments (55 g weighted). For the “Biotic H-cell” reactor, the bio-cathode was filled with 0.200 L of inoculum, and TCE was added as an electron acceptor. On the other hand, the anode consisted solely of the mineral medium solution (0.200 L). A schematic draw of the experimental configuration is shown in Figure 2. In comparison, the “Abiotic H-cell” reactor was built using the same configuration; however, the anode and cathode chambers were filled with 0.200 L of mineral medium solution and contaminated (no inoculum in the cathodic chamber). Both the H-cell systems were connected to a VSP300 potentiostat (biologic) and were operated with the cathode polarized at −0.9 V vs. SHE (i.e., −1.1 V vs. AgACl). The sequential fed-batch condition was maintained weekly, for the cathodic chamber only, following the same “Fill&Draw” procedure described above. The solution in the anodic chamber was replaced with a fresh mineral medium.

### 2.4. Analytical Methods

The formation of TCE reduction products was monitored by daily sampling the headspace of lactate-fed, H_2_-fed, and biotic H-cell reactors. As no TCE was spiked in the anode compartments and the abiotic H-cell, these chambers were only monitored once per week. 50 μL of gas-phase was sampled with a HAMILTON^®^ (Reno, NV, USA) gastight syringe and directly injected into the Gas Chromatograph.

CAHs, ethylene, and ethane were determined with a (GC) DANI MASTER^®^ (capillary column 30 m × 0.53 mm ID × 2.65 μm), with a flame ionization detector (FID) (DANI Instruments, Contone, Switzerland). The conditions were: He as carrier gas (flow 35 mL/min); 180 °C injector temperature; 200 °C detector temperature with H_2_, N_2_, air (flows 25, 25, 200 mL/min). The oven temperature was programmed as follows: 50 °C for 2 min; 20 °C/min and 210 °C for 5 min. 

With a second sampling, the H_2_, O_2_, and CO_2_ were also determined using a (GC) DANI MASTER^®^ equipped with a Thermal Conductivity Detector (TCD). Instrumental conditions were: 50 µL injection volume, N_2_ as a carrier gas, 12 mL/min flow, 120 °C injector temperature, 70 °C column temperature, and detector at 150 °C.

### 2.5. Data Elaboration

For each set-up, the rate for the RD reaction (RD rate) was calculated according to Equation (1). The RD rate represents the amount of equivalent required for the TCE biodegradation, in which trichloroethylene [TCE], cis-dichloroethylene [cis-DCE], vinyl chloride [VC], ethylene [Eth], and etane [Eta] are the nominal concentration (Cn) expressed in mM, while 2, 4, 6, 8, or 10 are the number of moles of electrons required for each reaction’s step. The coulombic efficiency (CE) of RD was calculated with Equations (2) and (3). The nominal concentration conventionally indicates the concentration of the compound as if it were all in the liquid phase (the calculation, therefore, takes into account Henry’s Constant of each compound) [37]:

RD rate (μeq/d) = (V liquid phase)/days {2[cis-DCE] + 4[VC] + 6[Eth] + 8[Eta]}(1)

RD (mA) = (RD rate (μeq/d) × 1000)/(s/d) F(2)

CE (RD) % = (RD (mA))/(I (mA)) × 100(3)

F indicates the Faraday constant (96.485 C/mol), s/d represents the 86.400 s in a day, and I is the current. Equation (3) represents in percent the quantity of electricity used by the reaction, expressed as CE (RD)%.

## 3. Results and Discussion

### 3.1. Lactate and H_2_ Fed Tests

**Figure 3:**
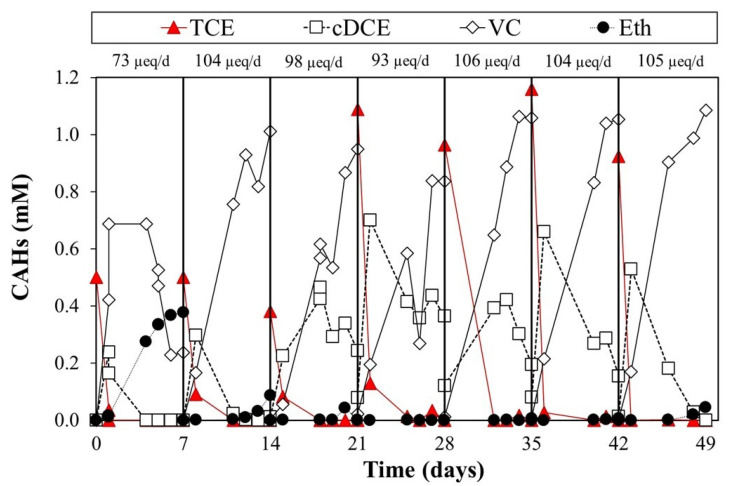
Lactate-fed reactor monitoring.

Figure 3 shows seven cycles of the lactate-fed reactor, which was adopted as a benchmark test of the experimental setup. Each cycle showed a quantitative conversion of TCE to the main reductive dechlorination products. TCE was fully converted into cis-DCE and VC. The RD rate, calculated on the RD intermediates, was constant for all the seven cycles, with an average value of 98 ± 4 µeq/d. Since the inoculum comes from biomass acclimatized to lactate, the experimental data confirmed the effectiveness of lactate as a hydrogen slow-release source through its fermentation. Nevertheless, seven working days were not sufficient to complete the conversion of TCE to non-toxic ethylene.

**Figure 4:**
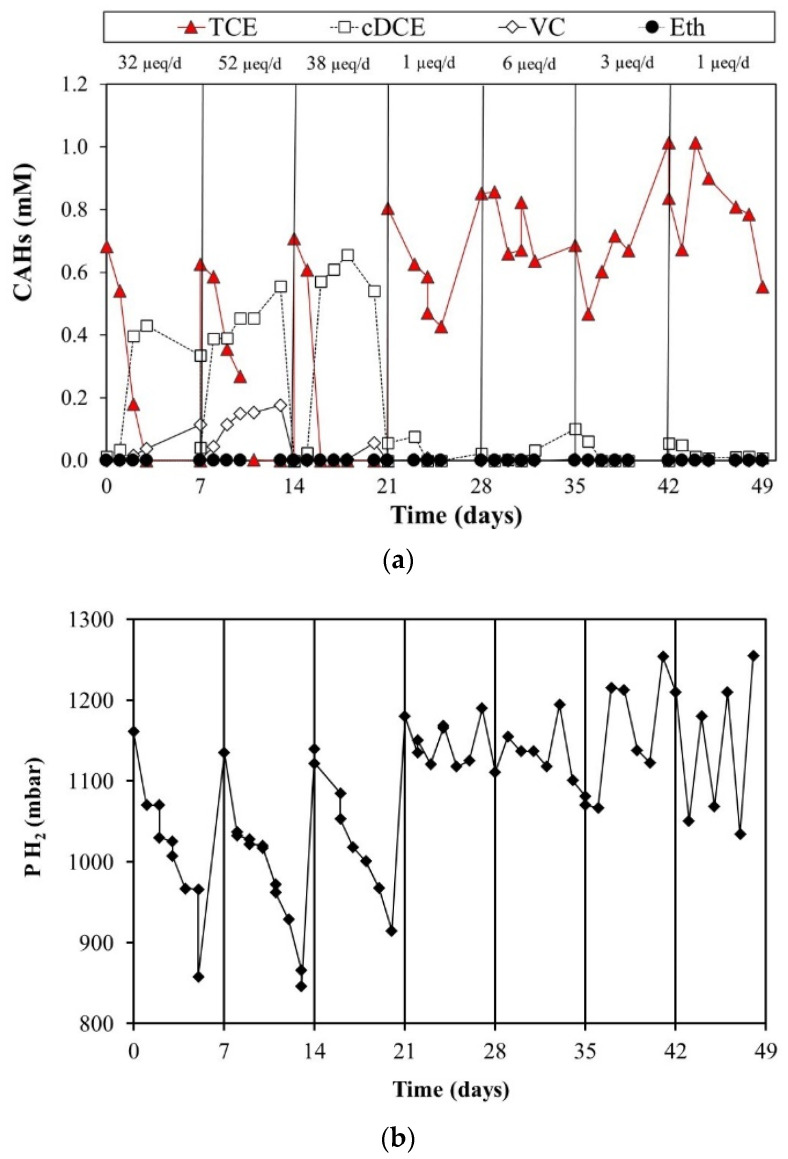
H_2_-fed reactor monitoring (**a**) and H_2_ partial pressure behavior over time (**b**).

On the other hand, the second batch was fed by molecular hydrogen as an electron donor. As reported in Figure 4a, during the first three cycles, TCE was significantly converted in cis-DCE and VC. Otherwise, starting from the fourth cycle, neither significative TCE removal nor intermediates formation was observed. Indeed, TCE concentration remained constant over the seven-day monitoring period, consequently causing a significant decrease in the RD rate. These results suggested a worsening of biomass performance with the consequent RD rate decrease, which was also confirmed by the hydrogen partial pressure over time (Figure 4b). The loss of dechlorinating activity in the H_2_-fed reactor was probably driven by the low solubility of molecular hydrogen; as a result, the electron donor was less available. In addition, a possible deficiency of growth factors and metabolite accumulation may have triggered this phenomenon against the *Dhc* consortium. For instance, the study performed by Di Stefano et al. (1992) predicted a different behavior of MeOH-fed and H_2_-fed cultures [38]. The PCE removal performance worsened for the H_2_-fed culture alone after a few cycles of activity. According to the authors, this was motivated by the lack of growth factors. On the contrary, the consortium was maintained under methanol as an electron donor did not suffer similarly. This assumption was further supported by Aulenta et al. (2005), who observed greater stability and long-term dechlorination activity in methanol and butyrate-fed bioreactors relative to the H_2_-fed bioreactor. This is explained by the presence of non-dechlorinating bacteria capable of creating optimal habitat for *Dhc* maintenance [35]. Furthermore, our findings are in good agreement with a recent study by Chau and colleagues, where *Dhc* strain CBDB1 (under lactate, propionate, or acetate feeding) showed higher activity and the RD rate was two or threefold higher in the presence of syntrophic partners than that of *D. mccartyi* pure culture (hexachlorobenzene as contaminant target). Notably, the authors reported that the co-presence of *Desulfovibrio vulgaris*, *Syntrophobacter fumaroxidans,* and *Geobacter lovleyi* supported H_2_, acetate, and cobalamin supply, also preventing the toxic effect of CO accumulation (resulting from acetyl-CoA cleavage) [39].

### 3.2. Performances of Biotic and Abiotic H-Cell Reactors

**Figure 5:**
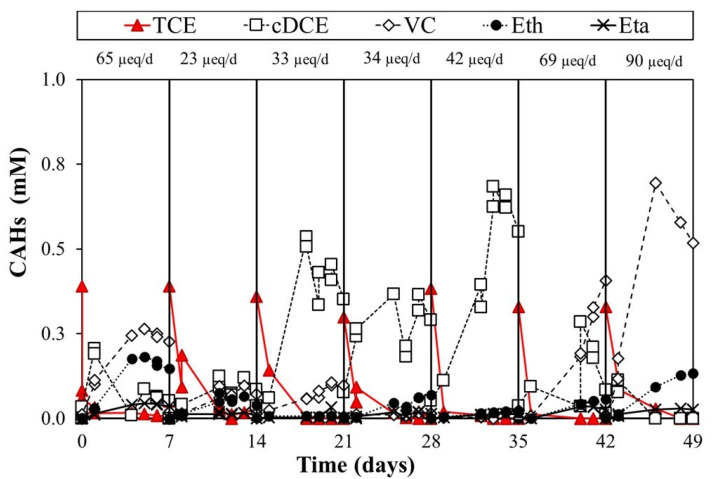
Bioelectrochemical system monitoring. The weekly cycles and RD rates (μeq/d) values, calculated according to Equation (1), are evidenced.

Figure 5 shows the RD time course of the Biotic H-cell reactor. In the bio-cathode, the total conversion of TCE was obtained in the first day of each cycle. The main RD by-products were cis-DCE and VC (notably in the last cycles). However, ethylene and ethane were also detected, even if in minor concentrations. This behavior reports the ability of the microbial consortium to fully degrade TCE even to not harmful products in seven working days, suggesting a more availability of the electrons to be used efficiently for the RD reaction. Moreover, this finding indicates that RD in the presence of lactate may be subject to some limitations due to the availability of hydrogen, hence the cycle time appears to be insufficient to provide the required hydrogen to achieve the reduction to ethylene and ethane.

**Figure 6:**
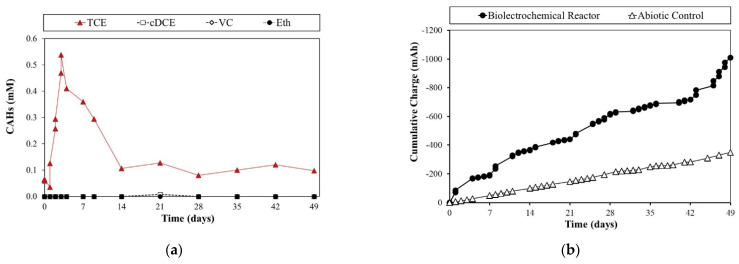
The CAHs monitoring in the abiotic H-cell system (cathode) (**a**) and the cumulative current (mAh) of the two electrochemical systems in comparison (**b**).

To exclude the presence of different TCE degradation pathways, an “abiotic H-cell” (no inoculum) reactor was performed by the polarization of the cell at the same cathodic potential of −0.9 V vs. SHE. As shown in Figure 6a, even if an almost constant TCE concentration was maintained during the first 5 days, a decrease in TCE concentration till the value of 0.1 mM was observed; the 0.1 mM TCE concentration remained constant for the rest of the operation period. The TCE decrease was probably due to sorption phenomenon that occurred on the graphite rod and granules which constituted the cathodic material. The complete absence of any dechlorination by-product clearly indicated the complete absence of any electrochemical degradation pathway on the abiotic cell. Moreover, Figure 6b showed a different behavior of the cumulative charge in the biotic and abiotic H cell. Indeed, the biotic H-cell reactor had a higher slope (i.e., the average current) with respect to the abiotic control. Since the only difference was the presence of the inoculum, this trend over time was caused by an increase in electron consumption for biological reduction reactions (translated into an increase in circulating current).

### 3.3. Comparative Evaluation of Different Electron Donors

To evaluate the efficiency of electron donor consumption for lactate-fed and H_2_-fed reactors, the following hypotheses were formulated:

- All lactate added to the culture was entirely fermented with H_2_.
- The bioavailable hydrogen that was entirely consumed by the RD reaction was the hydrogen dissolved in the liquid phase.

**Figure 7:**
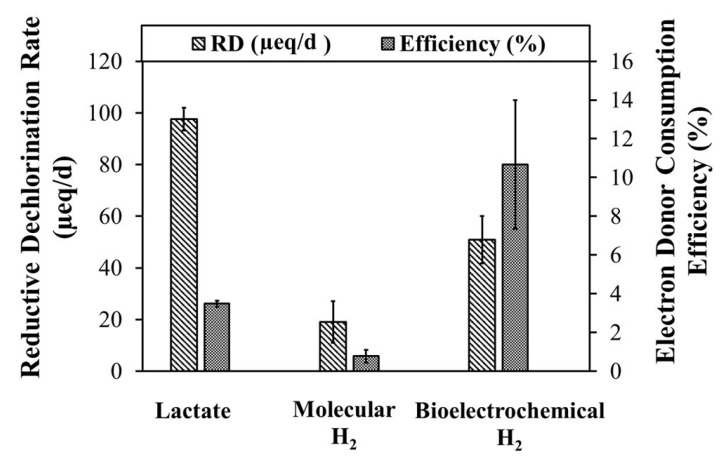
RD reaction rate and percentage efficiency of the electron donor consumption for the investigated systems.

As shown in Figure 7, RD rates and efficiencies calculated for each bioreactor were found to be significantly different, although the final electron donor is always the molecular H_2_. Globally, lactate-fed culture had the highest TCE elimination kinetics, with a higher value for RD (98 ± 4 µeq/d. This is likely due to the higher calculated values of mass recovery associated with the presence of a highly selected consortium, consisting mainly of *D. mccarty* (more than 70% reads were associates at *D. mccarty*). On the other hand, in terms of effectiveness, considering the maximum theoretical production of H_2_ by lactate fermentation, only 3.5 ± 0.2% was gained. The H_2_-fed culture, as already reported, removed TCE in only the first three cycles, losing over time the ability to perform the RD22. This negatively affected the rate and efficiency, compared to the other batch reactors, with 19 ± 8 µeq/d and 0.8 ± 0.3%, respectively. Furthermore, this result suggests a lack of interaction between the microbial consortium and dissolved molecular hydrogen. As a result, the electron donor was not fully bioavailable. It may also be influenced by the fact that the system was not agitated, thereby reducing gas transfer in the liquid phase. However, this configuration was required to compare performance with the BES system, which cannot be stirred. The H-cell bioreactor had half the kinetics of the lactate reference reactor (RD rate 51 ± 9 µeq/d concerning to 98 ± 4 µeq/d), otherwise, the Coulombic efficiency (calculated as the amount of current consumed for the reaction that takes place on the working electrode in comparison with the total amount of flowing current) reported a major value of efficiency (10.7 ± 3.3%). This is evidence of a more effective supplying electron donor of BES, consistent with other bioelectrochemical applications [36].

### 3.4. Outlook and Perspectives of the Study

The experimental results of the study showed the potential advantage of the bioelectrochemical approach in terms of the efficiency of electron donor supply (even if the RD rate calculated is lower than the one sustained by the lactate fermentation). More specific considerations can be developed by examining real-world scenarios, in which lactate was adopted to stimulate the RD reaction. In the case study reported in a technical report (TR-2250-ENV) [40], a source zone contaminated with TCE and cis-DCE, with a concentration of 386 and 106 µg/L, respectively, was treated by using lactate for the stimulation of the indigenous microbial dechlorinating activity. In almost 3 years of activity, 13,154 kg of lactate has been injected by an injection well to remediate an estimated groundwater volume of 113,267 m^3^ (see Table 1). Comparing the theoretical electron equivalent request for the complete TCE and cis-DCE reduction to ethylene and considering the theoretical ratio of 6 moles of H_2_ per mole of lactate, the estimated electron donor efficiency of lactate resulted in 0.2%. A possible alternative would be the use of hydrogen produced by the electrolysis of water and supported by renewable energy sources. An assessment of the energy consumption of this method depends on the industrial cost of hydrogen production (4.5 kWh/m^3^ H_2_ [41]). Considering the above-mentioned case study, the H_2_ supply by electrolysis of water required an estimated energy consumption of between 0.01 and 0.1 kWh/m^3^, which is attractive from an energetic point of view (details in Table 2). Some considerations concerning the efficacy of the BES in the stimulation of the RD reaction can be made through the analysis of some literature studies, summarized in Table 3. Since there is a lack of BES large-scale applications reported in the literature and technical reports, good results are achieved for performance in laboratory-scale experiments [42]. As reported in Table 3, for the specific CAHs remediation purpose, the coulombic efficiency obtained by different authors ranged between 4 and 90%, depending on the adopted operating condition. It is important to point out that for the BES, the energy consumption and efficiency of the electron donor supply strongly depend on the reactor configuration and conditions. Generally, these involve the polarization strategy (i.e., potentiostatic, galvanostatic, two or three electrodes configuration), the hydraulic retention time, the electrolyte composition, and the presence of side reactions.

## 4. Conclusions

The study reports the comparison between three different electron donors for the reductive dechlorination of TCE from the same active dechlorinating inoculum. While the lactate-fed reactor (benchmark) reported good performance and higher RD rates, r was well-suited to other authors’ findings, as it did not maintain high dechlorination rates. the H_2_-fed reactor showed a significant dechlorination activity for only three cycles, but did not maintain high dechlorination rates, subsequently a sort of inhibition with interruption of degradation of the high chlorinated contaminant. Consequently, the hydrogenophilic test showed the lowest value for both RD rate and efficiency of electron donor utilization (19 ± 8 µeq/L and 0.8 ± 0.3%). On the other hand, the bioelectrochemical dechlorination test showed an intermediate performance in terms of dechlorination rate, with an overall dechlorination rate 48% lower (−0.9 V vs. SHE as cathodic potential), compared to the traditional lactate approach. However, the bioelectrochemical approach showed a higher electron donor efficiency with an average coulombic efficiency of 10%. This batch reactor showed a good reductive dechlorination reaction rate, 51 ± 9 µeq/L, coupled with the best value of efficiency 10.7 ± 3.3%, compared to the benchmark batch reactors. Future developments of these setups should also involve the use of biobased activated carbon to combine conductivity and adsorption ability.

## Figures and Tables

**Table 1:** Stimulation of the RD activity by lactate in a case study ^1^.

Bioremediation by Injection of Lactate ^1^	
TCE and cis-DCE source zone concentration (µeq/L)	22
H_2_ from lactate fermentation (molH_2_/mol OA)	6
Lactate injected kg/m^3^	0.12
Lactate efficiency (%)	0.14

^1^ Values calculated by [40].

**Table 2:** Energetic cost of H_2_ sparging approach for the evaluated case study ^1^.

Hydrogen Bioremediation Evaluation	
TCE and cis-DCE source zone concentration (µeq/L)	22
H_2_ energetic cost (electrolysis) (kWh/m^3^ H_2_)	4.5
H_2_ for complete RD (m^3^H_2_/m^3^GW)	0.0003
Minimal energetic cost of the remediation (kWh/m^3^GW)	0.001
Efficiency factor for H_2_ sparging	0.1–0.01
Estimated energetic cost of the remediation (kWh/m^3^GW)	0.01–0.1

^1^ Case study reported in [40].

**Table 3:** The efficiency of BES in the stimulation of reductive dechlorination of certain Chlorinated Aliphatic Hydrocarbons (CAHs).

Target Compound	BES Configuration	RD Coulombic Efficiency (%)	Ref.
PCE	Tubular membrane-less	22	[29]
PCE—1,2 DCA	Two Chamber/CEM	80.4–90	[43]
cis-DCE	Two Chamber/Nafion^®^	60–90	[44]
TCE-Cr (VI)	Two Chamber/Nafion^®^	4.66	[45]
TCE	Two Chamber/Nafion^®^	4.73	[23]

## Data Availability

Data available on request due to restrictions eg privacy or ethical.

## Abbreviations

MEC	microbial electrolysis cell
BES	bioelectrochemical systems
RD	reductive dechlorination
TCE	trichloroethylene
cis-DCE	cis-dichloroethylene
VC	vinyl chloride
Eth	ethylene
Eta	ethane
